# Objective interpretation of ultraviolet-induced luminescence for characterizing pictorial materials

**DOI:** 10.1038/s41598-023-47006-x

**Published:** 2023-11-19

**Authors:** M. Caccia, S. Caglio, A. Galli

**Affiliations:** 1 IBFM-CNR, Via Fratelli Cervi 93, Segrate, MI Italy; 2 Dipartimento Di Scienza Dei Materiali, Università Degli Studi Di Milano-Bicocca, Via Roberto Cozzi 55, Milan, Italy

**Keywords:** Imaging techniques, Characterization and analytical techniques, Applied physics

## Abstract

Ultraviolet-induced Luminescence (UVL) is the property of some materials of emitting light once illuminated by a source of UV radiation. This feature is characteristic of some mediums and pigments, such as some red lakes, widely used for the realisation of works of art. On the one hand, UVL represents a like strike for a researcher in the cultural heritage field: in fact, UVL allows to characterise the state of conservation of the paintings and, in some cases, to recognize at glance some of the materials used by the artists. On the other hand, the contribution of UVL to the study of the artefacts is almost always limited to qualitative observation, while any speculation about the cause of the luminescence emission relies on the observer’s expertise. The aim of this paper is to overcome this paradigm, moving a step toward a more quantitative interpretation of the luminescence signal. The obtained results concern the case study of pictorial materials by Giuseppe Pellizza da Volpedo (1868–1907, Volpedo, AL, Italy) including his iconic masterpiece *Quarto Stato* (1889–1901), but the method has general validity and can be applied whenever the appropriate experimental conditions occur. Once designed an appropriate set-up, the statistical comparison between the acquisitions performed on *Quarto Stato*, on a palette belonged to the master, on drafts made by the author himself and on a set of ad hoc prepared samples both with commercial contemporary pigments and prepared with the traditional recipe, shed some light on which materials have been employed by the artist, where they have been applied and support some intriguing speculations on the use of the industrial lakes in the *Quarto Stato* painting.

## Introduction

Ultraviolet-induced Luminescence (UVL), i.e. the property of some materials to emit light once illuminated by a source of ultraviolet radiation (UV), can be effectively exploited in different research^1–4^ and the study of cultural heritage artefacts, in particular paintings, does not make an exception^5–7^. This is not surprising since some mediums, such as polymerized oil and shellac^8,9^, or pigments, such as zinc or lead white and some red lakes^10–12^, display UVL. Moreover, partly due to the availability of low-cost portable equipment, partly because the induced luminescence can often be appreciated by the naked eye, UVL offers the possibility for characterising the state of conservation of the works of art and, in some cases, recognizing at glance some of the materials used by the artists. The literature in the field reflects these facts offering a great number of papers including ultraviolet-induced luminescence as an investigation method^9,12–16^. However, the contribution of UVL is almost always limited to qualitative considerations, while any speculation about the cause of the luminescence emission relies on the observer’s expertise. The present paper attempts to overcome this paradigm, moving a step toward a more quantitative interpretation of the luminescence signal. The proposed pioneering approach stems from three methodological pillars: a low-cost home-designed appropriate experimental set-up based on portable equipment, a consistent and reliable materials database, and a computer-driven protocol to manage and analyse the datasets. We designed this experimental approach as we found ourselves facing the challenge of studying the materials used by Giuseppe Pellizza da Volpedo in his worldwide famous masterpiece *Quarto Stato* (Galleria di Arte Moderna—GAM, Milan, Italy) (http://www.gam-milano.com/it/mostre-ed-eventi/il-quarto-stato/). In particular, the attention of art scholars focused our attention on the use of red lakes and on the relationship between the masterpiece and the introduction of the industrial pigment materials.

The experimental set-up is based on a hyperspectral camera, device designed for monitoring the reflectance of the surfaces in the 400–1000 nm range^17^, adapted for detecting UVL. This is possible because the luminescence of red lakes falls in a wavelength window (> 550 nm) compatible with the high efficiency detection range of the camera. Therefore, the emission spectra, characterised by well-defined bands, can be used as quantitative observables for understanding UVL. A set of such spectra can constitute a database that, if carefully tailored, can describe the samples under investigation. This paper shows how some ad-hoc prepared drafts have been designed, characterised, and prepared for generating a group of spectra that could reliably resemble the features of the materials expected to be present within the items by Pellizza. Even if the results cannot completely exhaust the description of the samples, they demonstrate the effectiveness of the approach in the considered case study. In fact, not only the presence of some kind of lakes on the surfaces of the samples has been confirmed or excluded, but it was also possible to obtain indications about the origin of the materials and support the discussion about the mutual interaction between pigments and binders. The fact that this work is focused on the artistic production of Pellizza da Volpedo is not a limit; the proof of concept behind the method has general validity and it is effective whenever the opportune experimental conditions occur, i.e. whenever it is possible to tailor a spectra database basing on the observation or the preliminary knowledge of the samples.

## Results

**Figure 1:**
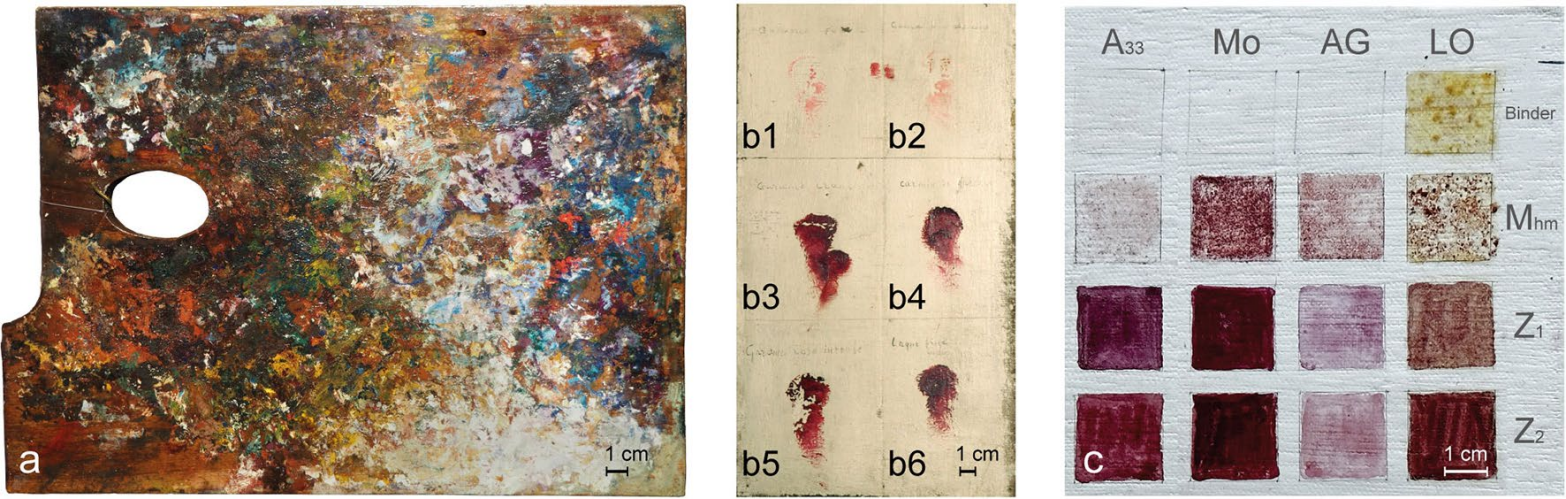
Visible light images of the various elements covered by the research, in (**a**) the wooden palette found in the painter’s studio, (**b**) some samples made with commercial pigments made by the artis himself, (**c**) test drafts carried out in the laboratory with four different binders (A_33_, Mo, AG and LO) and three different lakes, one produced in the laboratory (M_hm_) and two commercial (Z_1_ and Z_2_).

To recognise the presence of materials by the emission spectrum of UV induced luminescence, the painting *Quarto Stato* by Pellizza da Volpedo (http://www.gam-milano.com/it/mostre-ed-eventi/il-quarto-stato/), a wooden palette (Fig. 1, panel a) and the drafts (Fig. 1, panels b1–b6), found in Pellizza’s studio and a set of drafts made in the laboratory by the authors of this work (Fig. 1, panel c) have been compared. If for the painting and the wooden palette the pigments are unknown, for the artist’s proofs we know they are colours marketed at the end of nineteenth century by Lefranc Bourgeois for which Pellizza has marked the corresponding name next to each draft. The laboratory drafts are made using both contemporary industrial pigments provided by Zecchi in Florence, Italy (named Z_1_ and Z_2_) and lacquers extracted directly from madder roots with a traditional recipe (indicated with M_hm_)^18^, spread using two classic binders, linseed oil (OL) and Arabic gum (AG), and two contemporary binders, one acrylic-based, Acril 33 (A_33,_) and one polyvinyl-based, Mowital (Mo).

### Creation of a spectra database

**Figure 2:**
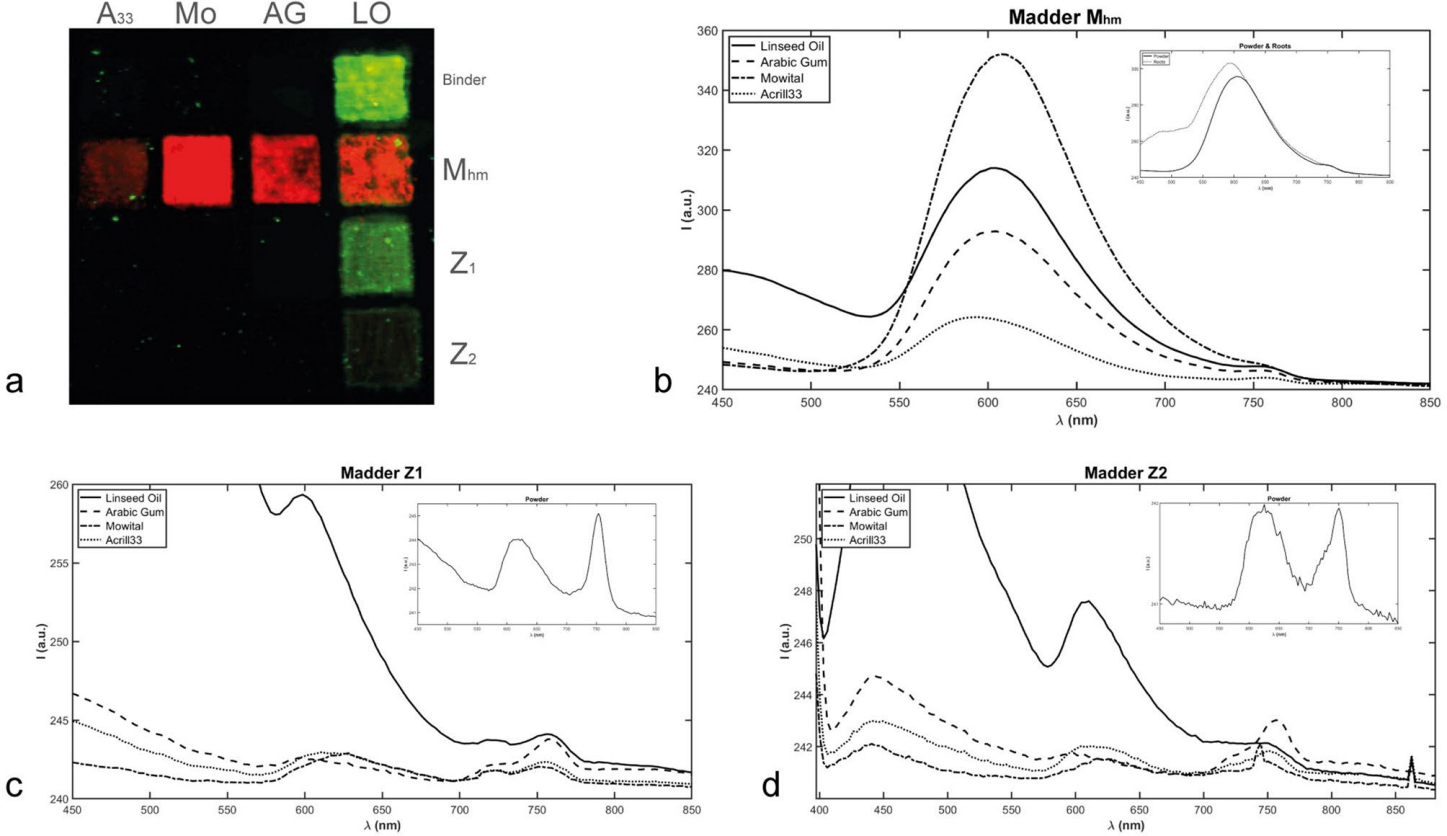
Image (**a**) of the UV-induced luminescence of the drafts prepared in the laboratory and the spectral emission signal (**b**) of the home-made madder lake, M_hm_, applied with the four binders and with the reference of the pigment powder and the roots, of the commercial (**c**) Z1 and (**d**) Z2 lakes, applied again with the four binders and with the reference of the unbound pigment powder.

The drafts prepared in the laboratory (Fig. 1, panel c) display the expected UV-induced luminescence emission (Fig. 2, panel a). The greenish signal from the drafts on the right side of the canvas is due to the well-known luminescence emission of Linseed Oil^19^ while the orange-red emission is related to the presence of madder in the pigments^13^. All the characteristic spectra for the 4 drafts containing the homemade madder pigments have an emission band around 600 nm; even if the maximum wavelength is not exactly the same ($\lambda$_maxMhm_ = 601 ± 5 nm), it is possible to argue that by changing the binder the main features of the UVL emission do not substantially change (Fig. 2, panel b). This conclusion is supported by the spectra of Fig. 2, panel b; they have been evaluated from hypercubes acquired on the powder and roots of the madder used to derive homemade based pigments and they are very similar to those of panel b. At the naked eye, the draft made with contemporary industrial pigments by Zecchi seem not to have luminescence emission at all, however, the characteristic spectra of the drafts highlight the presence of a weak emission band in the red region of the spectrum (Fig. 2, panels c and d). As in the case of the M_hm_ drafts, the position of maximum does not significantly change on varying the binder. With the exception of the drafts employing the Arabic gum as binder that have $\lambda$_maxZi_ < 600 nm, the wavelengths corresponding to the emission maxima of the others drafts are located near 610 nm ($\lambda$_maxZ1_ = 609 ± 14 nm and $\lambda$_maxZ2_ = 609 ± 10 nm) and they are slightly red-shifted with respect to their M_hm_ counterparts. The red shift of the emission band for both Z_1_ and Z_2_ has been also observed for the powders used to create the drafts (Fig. 2, panels c and d) confirming the weak emission band around 610 nm as a characterising feature for the Zecchi’s pigments. Surprisingly, Arabic Gum seems to have the opposite effect on the UVL of contemporary industrial pigments which results in blue-shifted emission with respect to their homemade counterparts. The reason for this behaviour deserves further investigation; however, for what concerns this work, the effect induced by AG on Zecchi’s pigments UVL has been accounted as pure experimental evidence. In order to confirm the possibility of comparing the drafts made in the laboratory with the artist’s original materials, it was also tested that the characteristics of the UV luminescence emission curves do not change by exposing the samples to photoaging in a solar simulator (see the Supplementary Materials for a more comprehensive treatment).

Note that, even in the case of the ad-hoc prepared drafts, for some polygons, the classifier could identify more than one characteristic spectrum; looking at Fig. 1, panel c and Fig. 2, panel a, this is not surprising due to the non-complete uniformity of the applications of the pigments. In these cases, the end member of the ROI chosen on the draft will be the characteristic spectrum obtained from that class that contains the largest number of elements. The other classes have been excluded by further analysis. The set of spectra resulting from the application of the classifier on the 12 ad-hoc prepared drafts constitute the database used to start to investigate the lakes of *Quarto Stato* painting and the other materials belonging to Giuseppe Pellizza da Volpedo.

### Comparing the database to the pictorial materials belonging to Giuseppe Pellizza da Volpedo

**Figure 3:**
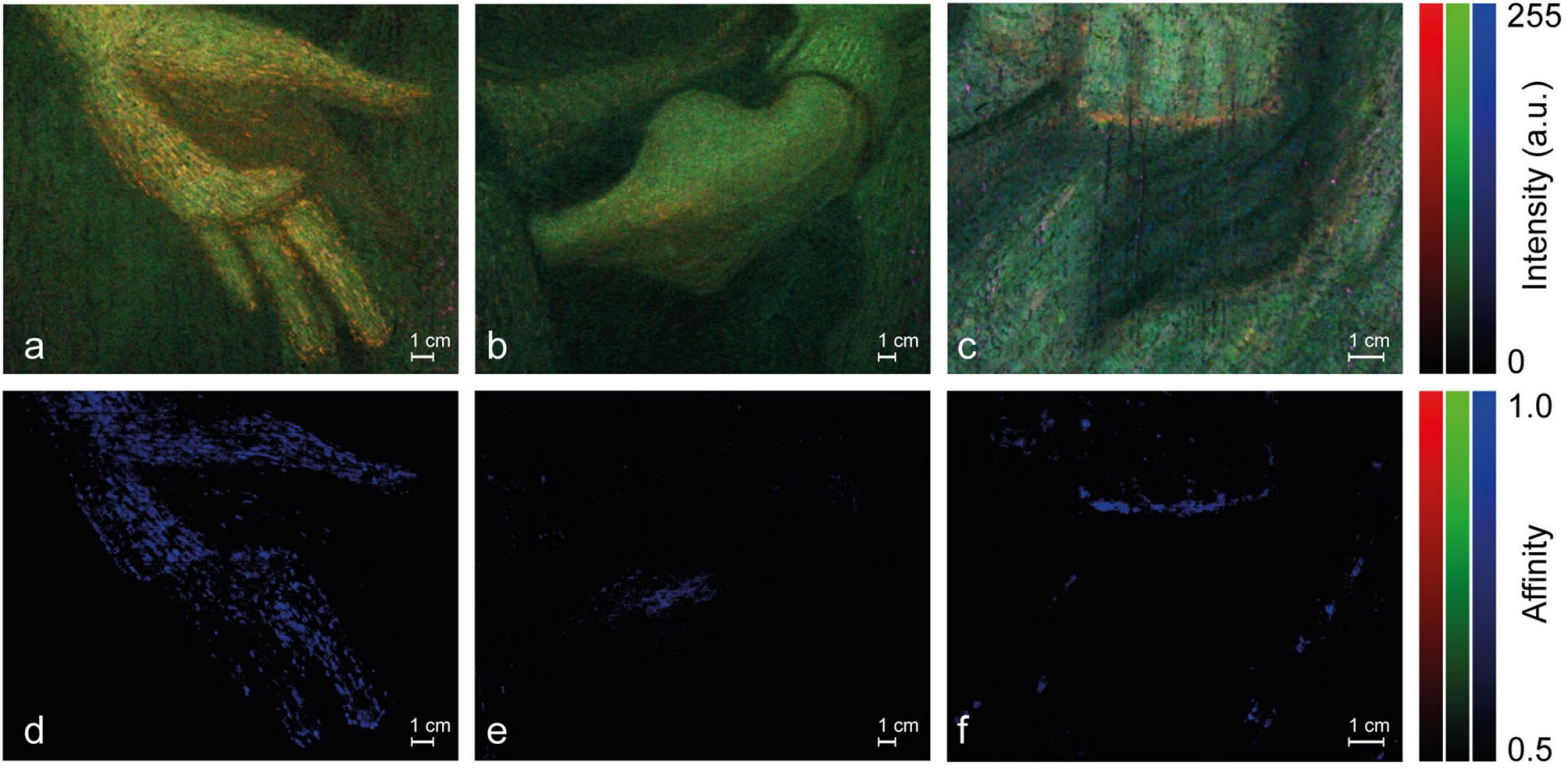
Top row: UV luminescence images of three details from the *Quarto Stato* painting: (**a**) the left hand of the woman in the foreground, (**b**) the left hand of the man in the centre foreground, and (**c**) the left foot of child held in the woman’s arms. In the lower row: RGB images composed by the affinity maps calculated between the hypercubes of the three details of the upper row (**d** the woman’s hand, **e** the man’s hand, **f** the child’s foot) and the lakes studied in the laboratory bound with Acril 33; in particular, in the red channel the map related to the comparison with Z_1_ is implemented, in the green channel the one referring to the comparison with Z_2_ and in the blue channel that of the madder lake M_hm_.

**Figure 4:**
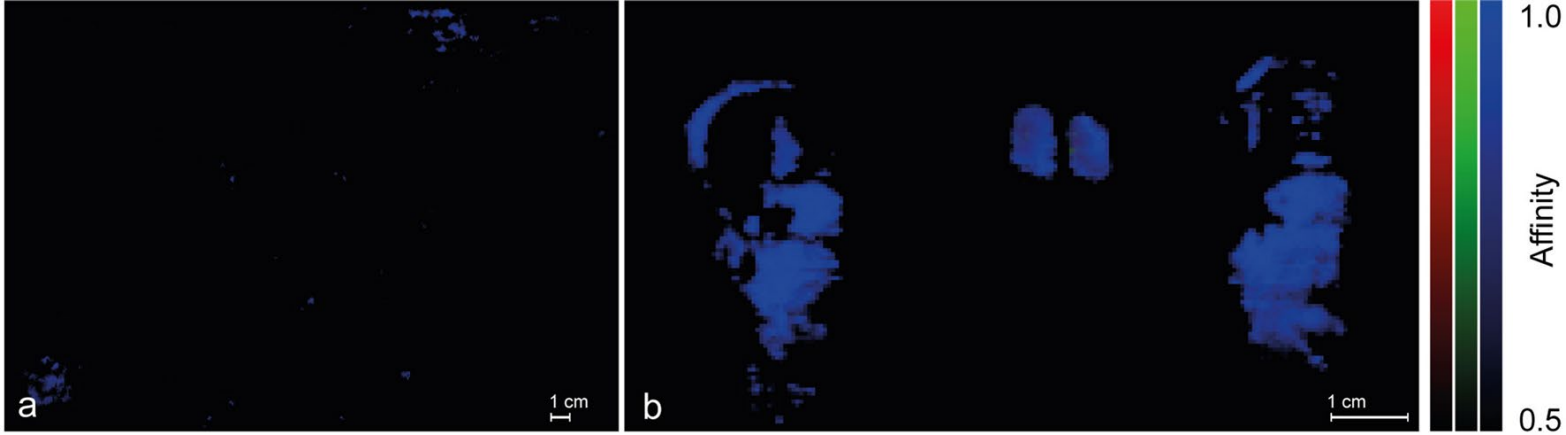
RGB images composed by the affinity maps between the lakes studied in the laboratory bound with Acril 33 and (**a**) the wooden palette and (**b**) two of the drafts found in the painter’s studio: Grance rose and Garance jaune capucine (ref. to Fig. 1, panels b1 and b2). In the red channel the map related to the comparison with Z_1_ is implemented, in the green channel the one referring to the comparison with Z_2_ and in the blue channel that of the madder lake M_hm_.

The database is constituted by the spectra reported in Fig. 2 that are representative of both the homemade and commercial madder pigments. The database spectra have been employed as endmembers (EMs) in the context of a spectral correlation mapper (SCM) and the degree of similarity between the database components and the hyperspectral data from the surface of the samples has been checked out. SCM is able to distinguish between correlated and anti-correlated objects^20^ because the similarity maps are matrices with values between 1 and − 1, where positive and negative values mean respectively correlation and anti-correlation. First row of Fig. 3 (panels a, b, and c) shows three areas of *Quarto Stato* painting where a significant UVL has been observed (refer to the orange tones in these three panels). The bottom row of Fig. 3 (panels d, e, and f) shows the RGB false colour images built by thresholding the similarity maps above 0.5 (i.e., high similarity) and merging together those referred to the EMs of the pigments created using A_33_ as binder (first column in Figs. 1, panel c and 2, panel a). The Red, r_ch_, Green, g_ch_, and Blue, b_ch_, channels correspond respectively to the thresholded SCM maps of lake Z_1_, Z_2_ and M_hm_. No reddish or greenish regions appear in the SCM maps, therefore it is straightforward to conclude that only the custom-produced pigment has a high affinity with the materials of the painting and to argue that the pigments applied on the canvas resemble the properties of the lake obtained following the traditional recipe rather than those industrially produced. Similar considerations, data not shown, can be done based on the RGB false colour images obtained using as endmembers the spectra from the drafts created employing Lo, Mo and AG as binders. The traces of, at least, a red lake very similar to the one produced following the traditional recipe are confirmed by comparing the database to hypercubes from the palette (Fig. 1, panel a) and a couple of drafts (Fig. 1, panel b). The blue regions correspond to areas with significant UVL signals and display high similarity with the EMs of M_hm_ (Fig. 4, panels a and b).

**Figure 5:**
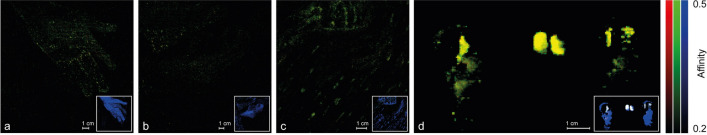
RGB images composed by the affinity maps with a lower threshold (0.2–0.5, i.e.: low affinity) between the lakes studied in the laboratory bound with Acril 33 and (**a**) the woman’s hand, (**b**) the man’s hand, (**c**) the child’s foot in *Quarto Stato* paining and (**d**) two of the drafts found in the painter’s studio: Grance rose and Garance jaune capucine (ref. to Fig. 1, panels c1 and c2). In the red channel the map related to the comparison with Z_1_ is implemented, in the green channel the one referring to the comparison with Z_2_ and in the blue channel (small inset) that of the madder lake M_hm_.

Lowering the threshold (0.2–0.5, i.e.: low affinity) the scenario for what concerns *Quarto Stato* painting does not change significantly. It is true that some reddish/greenish areas appear in the related channels (Fig. 5, panels a, b, and c) but they are almost superimposed on the blue regions of the high-affinity maps (Fig. 3, panels d, e and f) rather than identify other parts of the painting observed areas. The areas of low similarity with pigments containing madder Z_1_ and Z_2_ are not surprising and can be ascribed to the fact that the raw material is always madder even if it undergoes industrial unknown protocols to obtain the final pigments. The previous speculation is supported by the maps obtained on the Pellizza’s drafts: those referred to Garance rose and Garance jaune capucine (two pigments containing madder^21^) display weak similarity with Zecchi’s pigments in the same areas characterised by high affinity with M_hm_ (Fig. 5, panel d).

**Figure 6:**
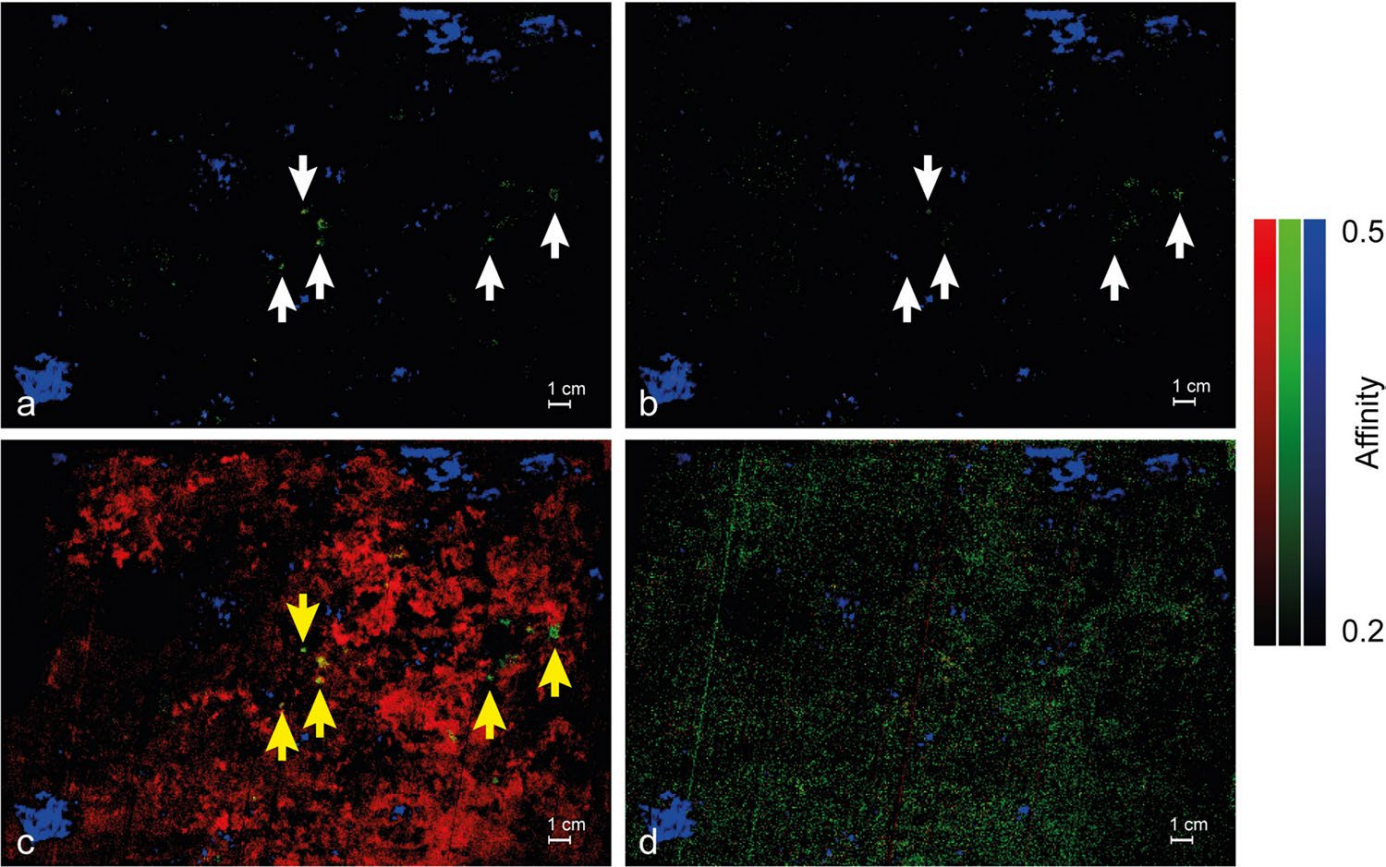
RGB images composed by the affinity maps with a lower threshold (0.2–0.5, i.e.: low affinity) between the wooden palette and lakes studied in the laboratory bound with (**a**) Mowital, (**b**) Acril 33, (**c**) linseed oil and (**d**) Arabic gum. In the red channel the map related to the comparison with Z_1_ is implemented, in the green channel the one referring to the comparison with Z_2_ and in the blue channel that of the madder lake M_hm_.

More entangled are the low-affinity SCM maps of the wooden palette (Fig. 6). On the one hand, the blue areas (b_ch_ in Panels a, b, c, and d) are the same as in Fig. 4, panel a and therefore they do not add information about the homemade madder distribution. On the other hand, the observation of the red and green channels deserves more attention because they reveal that the low similarity maps linking Zecchi’s madders to the palette show details depending on both the binder and the lake. The maps of the pigments where Mowital (Mo) and Acril 33 (A_33_) have been employed as binders highlight weak spots in the green channel (g_ch_) superimposed to even more weak ones in the red channel, r_ch_ (white arrows in Fig. 6, panels a and b). Even if the similarity is low, these spots appear significant because, differently from the cases of *Quarto Stato* and of the author’s drafts, they identify regions of the palette which are unrelated to M_hm_. The green and red channels of pigments with Linseed Oil (Fig. 6, panel c) display a second interesting issue: the behaviours of Z_1_ and Z_2_ are different from each other and, while g_ch_ identifies the same isolated bright spots of panels a and b (yellow arrows in Fig. 6, panel c), r_ch_ is dominated by a largely diffused unspecific low similarity. This is quite unexpected because looking at the profiles of the endmembers (continuous lines in Fig. 2, panels c and d), in the 550–730 nm range the end members differ only for the relative intensity of the characteristic emission band (the secondary maximum near 610 nm on the right side of the luminescence peak due to the linseed oil, LO) which in the case of Z_1_ is less prominent than in that of Z_2_. Anyway, it seems unlikely that such a small difference should account for what is shown in panel c of Fig. 6; it is more probable that the cause of these facts can be found in the properties of industrial contemporary lakes provided by Zecchi and their interactions with those of the binder. The green and red channels of pigments bound with Arabic gum (Fig. 6, panel d) do not evidence any area of similarity at all. This is another interesting but less surprising issue; the fact that Z_1_ and Z_2_ mixed to AG display a singular behaviour, adds to the evidence that the characteristic emission of both the pigments is blue- rather than red-shifted in comparison with that of their homemade counterpart and gives further support to the idea that Arabic Gum could play a role in the determination of the UVL features of the commercial lakes. Taken together, these experimental results ensure that there exists more than one kind of madder lake within the materials by Pellizza and, more importantly, demonstrate that the current database can, at least partially, help to hint at the presence of some pigments and exclude that of others even if the spectral difference is not evident at glance.

### Deepen the comprehension of the pictorial materials belonging to Pellizza da Volpedo

**Figure 7:**
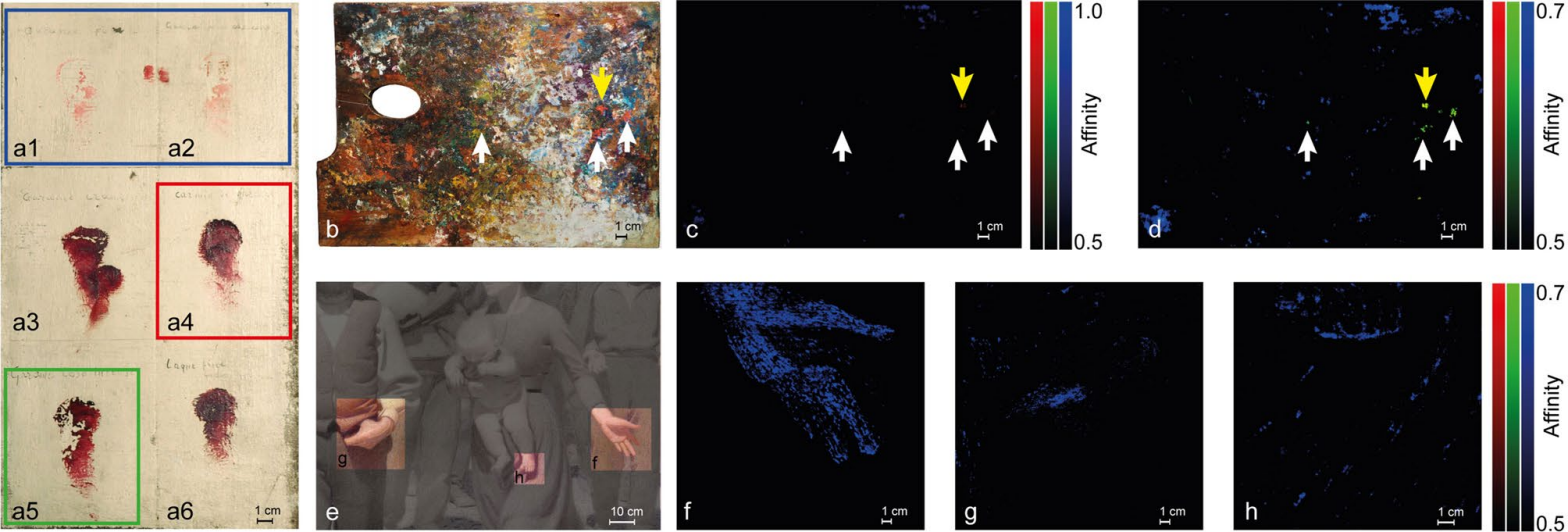
Comparison between (**a**) the drafts found in the painter’s studio, (**b**) the wooden palette and (**e**) the *Quarto Stato* painting. The luminescence spectra from drafts made by the artist were used as endmembers for the creation of the affinity maps with which the false colour images of the (**c**,**d**) wooden palette and (**f**,**g**,**h**) details of the painting are composed. In the red channel the map related to the comparison with (**a4**) Carmin de garance is implemented, in the green channel the one referring to the comparison with (**a5**) Garance rose intense and in the blue channel that of (**a1**) Grance rose and (**a2**) Garance jaune capucine.

The abundance of materials belonging to Pellizza and held in the studio museum are alike strike since they give a double opportunity: on the one hand, they can be used for completing the depicted drawn by means of the database created in the laboratory; on the other hand, the autograph annotations left by Pellizza below the drafts allow to deepen the discussion about the lakes that have been supposed suitable for realising his works. Panel a of Fig. 7 assigns a false colour to the author’s drafts that have been found in the palette and in *Quarto Stato* by SCM analysis. Garance rose and Garance jaune capucine have been grouped into a single channel (the blue one) because the classifier identifies both these drafts by the same end member. Carmin de garance and Garance rose intense have been assigned to the red and green channels respectively, while Garance cramoise and Laque fine de Garance Adrinople have been left blank because neither the palette nor *Quarto Stato* painting show significant affinity with their end members. Panels b, c and d and panels e, f, g and h of the same Figure illustrate the results of SCM analysis involving the drafts highlighted in panel a and, respectively, the palette and *Quarto Stato*. The blue spots in panels c, f, g, and h hint at the presence of the couple Garance rose/Garance jaune capucine on the surfaces of both the palette and the painting. This is not surprising because the drafts of these lakes show high affinity with M_hm_ based pigments, i.e. the only database pigments that display a high affinity with the palette and the painting (Figs. 3, 4). Coherently with these findings, the blue spots in the panels of Figs. 3, 4 (panel a), and 7 identified the same areas. In summary, it is possible to argue that (i) only Garance rose and Garance jaune capucine can be found in *Quarto Stato* and (ii) if Garance rose and Garance jaune capucine are commercial, they are more similar to lakes obtained by the original recipe than those obtained by purchased products.

Panel b of Fig. 7 has another outstanding feature: it shows a faint red spot corresponding to a red hue in the palette (yellow arrow in Fig. 7 panels b, c and d). Lowering the upper limit of the SCM map (Fig. 7, panel d) the blue spots saturate (as expected) and, more interestingly, one green and a few other red/green spots become evident (white arrows in panel d). These small regions are reach of meaning. Firstly, they prove that (i) there exist at least two more lakes within the material chosen by Pellizza for his own palette and (ii) these lakes can be found alone or mixed in the palette but (iii) they have not been used in *Quarto Stato*. Moreover, since all the spots indicated by the arrows in panel d of Fig. 7 find a counterpart with the spots identified by the commercial lakes in Fig. 6, it is possible to ensure that, within the lakes classified and selected by Pelizza, Carmin de garance and Garance rose intense can be rated commercials.

**Figure 8:**
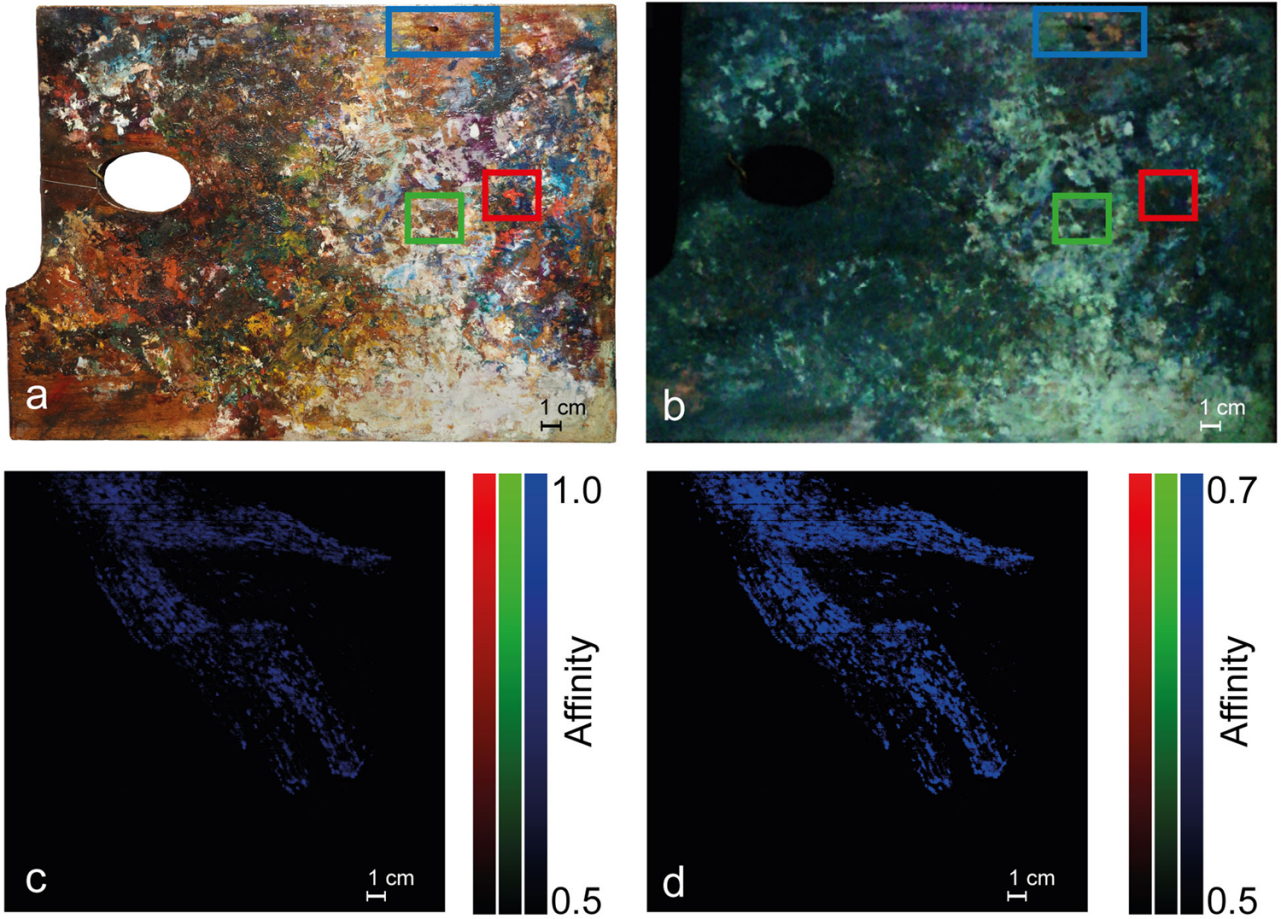
Comparison between the luminescence spectra identified on the wooden palette and the detail of the woman’s hand in the *Quarto Stato* painting. In the (**a**) visible light and (**b**) UV-induced luminescence images, the areas from which the luminescence spectra used as endmembers in the creation of the (**c**,**d**) similarity maps were extrapolated are highlighted.

Figure 8 summarises the SCM output obtained comparing the endmembers corresponding to the areas of the palette where the traces of lakes ascribable to the couple Garance rose/Garance jaune capucine, Carmin de garance and Garance rose intense are put in evidence (coloured squares in panel a; the inset of panel a recap the location of the areas within the palette) with *Quarto Stato*. The similarity maps, (Fig. 8, panels c and d) support the situation depicted above only lakes that are very similar to both the couple Garance rose/Garance jaune capucine and the madders obtained following the original recipe, have been surely involved in the realisation of *Quarto Stato* painting.

## Discussion

The spectra database built in this work is limited to red lakes present in an original wooden palette of masterpiece Giuseppe Pellizza da Volpedo (Fig. 1, panel a), in some draft prepared by Giuseppe Pellizza da Volpedo (Fig. 1, panel b) and it is based on the ad-hoc prepared drafts (Fig. 1, panel c). The simultaneous presence of Zecchi commercial pigments (Z_1_ and Z_2_) and home-made madders (M_hm_,) mixed with traditional, Arabic gum (AG) and linseed oil (LO), and modern binders, Mowital (Mo) and Acril 33 (A_33_), accounts for the facts that *Quarto Stato* painting dates back to 1898–1902 and that it collocates in a period during which handmade pigments started coexisting with industrial products. First of all, it is interesting to note that, despite the low number of laboratory references (Fig. 1, panel c), it is possible to distinguish the different lakes and the different binders (Fig. 2). The high-affinity maps involving the materials belonging to the author (Figs. 3, 4) show that only the database pigments containing homemade madder (M_hm_ in the blue channel) display high similarity with the luminescent areas of the samples disregarding the binder employed to create the drafts. This could suggest that the products commercialised at the beginning of the last century and employed in the painting were not so different from the handmade ones. Lowering the affinity threshold does not change the scenario (Fig. 5).

Moving the attention to the Spectral Correlation Mapper analysis (SCM) performed comparing each other the materials belonging to Pellizza, it is possible to individuate, with great confidence, which kind of lakes, between that experienced by the author and classified in the drafts of Fig. 1, panel b, have been selected for his palette and used in *Quarto Stato*.

When the author drafts are considered as EM to characterise the pigments present in the painter’s wooden palette and in *Quarto Stato* painting (Fig. 7), the low-affinity maps exclude certain types of commercial products, namely Garance cremoise, Carmin de garance, Garance rose intense and Laque fine Garance Andrinople (Fig. 7, panels a3–a6). Moreover, traces of three of these lakes are individuated in the wooden palette of the Master (Fig. 7, panels c and d), namely the couple Garance rose (a1)/Garance jaune capucine (a2) in the blue channel, Carmin de garance (a4) in the red channel and Garance rose intense a5) in the green one. Differently, only the couple Garance rose/Garance jaune capucine has been found in *Quarto Stato* (Fig. 7 panels f–h). These results are confirmed by the comparison between the EMs of the wooden palette areas containing traces of the three lakes mentioned above and the painting (Fig. 8). All this evidence demonstrates that, within the lakes tested on the drafts by Pellizza and effectively founded in his wooden palette, only the couple Garance rose/Garance jaune capucine has been surely employed in *Quarto Stato*.

It must be into account that if the luminescence band ascribable to the binders is predominant with respect to the bands related to the pigments themselves, the SCM is not able to distinguish the pigments (Fig. 6).

## Conclusion

In this paper we have shown that it is possible to move beyond the use of UVL as a qualitative tool. The proposed approach built around the UVL hyperspectral data acquired by adapting the Specim IQ reflectance detection system represents a quantitative method for studying samples of historical interest. In the case of the considered red lakes, the results demonstrate that once the ad-hoc created drafts in the right conditions, their ultraviolet-induced luminescent spectra were suitable to create a database supporting the analysis of materials used in the real cases under investigation: the database helped to support or exclude the presence of specific kinds of pigments. Moreover, these goals have been achieved disregarding the expertise of the observer and basing only on the statistical analysis of the detected hyperdata. It is true that the availability of several materials belonging to the master is not common and that some aspects highlighted by the presented approach, such as the role of the pigment-binders interactions, deserve further investigation, but the possibility for quantitatively exploiting the UVL signal has been definitively proven. By checking the presence of the material used in the original draft by Pellizza in the wooden palette and in *Quarto Stato*, it was possible to achieve two goals: first of all, the test of goodness of the methodological approach and, more importantly, the map of a specific presence of the red lake in *Quarto Stato*. Finally, the fact that the present database concerns the specific case study of Pellizza da Volpedo cannot be considered a real limitation because the approach has general validity and can be applied whenever the appropriate experimental conditions occur, particularly in the case of contemporary artworks in which similar supplies of materials can be found^22^.

## Materials and methods

### Quarto Stato painting

*Quarto Stato* (1898–1902 ca.) by Giuseppe Pellizza da Volpedo (http://www.gam-milano.com/it/mostre-ed-eventi/il-quarto-stato/), is a famous oil on canvas held in the “Galleria d’Arte Moderna—GAM” in Milan. This big painting (283 × 550 cm) is the final outcome of a creative process that lasted ten years, ending after its first exhibit at the Quadriennale in Turin in 1902. Acquired by the City of Milan in 1922 via a public petition. Due to the divisionist technique employed by the Master, the shades perceived by the observer’s eyes are the result of an intertwined set of brushstrokes of different pigments adapted by Pellizza to his perception of colour theory^23,24^. This fact makes the determination of the materials used by the artist a challenging task; however, it is well-known that Pellizza used some red lakes that under UV excitation give rise to a luminescence signal in the orange-red region of the electromagnetic spectrum^13,22^; the identification of these pigments is a suitable task for the present experimental approach.

### Wooden palette

A palette belonging to Pellizza da Volpedo (Fig. 1, Panel a) is one of the objects held in the Studio Museum located in the birthplace of the master (Volpedo, AL, Italy) and made available for this research. As easily predictable, does not exist exhaustive documentation about the palette and it looks exactly like everyone can expect: a disordered mix of different shades superimposed and flanked without a clear rational scheme. However the art experts collocate this object in a time range comparable to that of the realisation of *Quarto Stato* painting even if they hypothesise that the masterpiece used it in the open air; therefore, even if it cannot be said that this is really the palette used for the painting, it is possible to suppose that it contains materials very similar to those of *Quarto Stato* making the palette an interesting item for the aim of this study.

### Master’s drafts

The Master’s drafts (Fig. 1, panel b) are a few small spots of colour made by Pellizza on some little canvas areas. Most of the considerations about the palette remain true for the drafts: a complete catalogue of these hues does not exist but, luckily, Pellizza took note of their names just above the spots making the association of every single draft to a commercial pigment form Lefranc & Bourgeois found in the studio of the master.

### Ad hoc drafts

The ad-hoc prepared drafts (Fig. 1, panel c) have been designed to study the lakes used by Pellizza da Volpedo when he realised *Quarto Stato*. On the one hand, the artist was known as one the first painters that experienced the use of industrial pigments, on the other hand, it is quite rational to suppose that the first commercial products could be more like the traditional hand-made pigments rather than to modern available production. Therefore, three madder lakes have been selected: two representing industrial production and provided by Zecchi store in Florence (called Z_1_ and Z_2_) and one synthesized in the laboratory directly from madder roots according to the traditional recipe (called home-made madder, M_hm_)^18^. The three pigments were spread into 12 small squares on a cardboard bound with 4 different binders: linseed oil (LO), Arabic gum (AG), Mowital (Mo) a vynilic based binder and Acril 33 (A_33_) an acrylic based binder. Other 4 squares were prepared with the binders alone to check their possible luminescence. The binders have been selected, on the advice of restorers, to cover the different possibilities available to artists, from the classic ones to the most modern and contemporary ones.

### Powders and roots

For a better comprehension of the luminescence characteristics of the lakes without the influence of other factors such as the binders or the substrate supporting the layers in drafts samples, the unbound powders and some pieces of madder roots were also examined. These samples were placed on special 3D-printed supports employing PLA plastic, a product that does not emit any luminescence when irradiated with UV sources.

### The imaging experimental set-up

The experimental set-up is based on the portable Specim IQ spectral camera^17^ by Specim Spectral Imaging LTD (Oulo, Finland). It employs the push broom principle measurements of a scene by scanning line-by-line (each line is made by 512 pixels) between 400 and 1000 nm^25^. The spectral information is collected at a resolution of 7 nm with 204 binnable bands across the wavelength range. The number of acquired lines is static and equal to 512 therefore the camera captures hypercubes constituted by sequences of square images of 512 × 512 pixels. The number of bands determines the third dimension of the hypercubes and it depends on the binning. The focusing of the camera is set by the user by manually adjusting the focus system and the camera-sample mutual distance. At 1 m, the viewable area is 0.55 × 0.55 m resulting in a resolution of 1.07 mm at the target. The Specim IQ is designed to collect reflectance data, therefore the acquisition set-up is usually completed by a lighting system of 2 halogen lamps arranged symmetrically with respect to the sample and oriented to ensure the correct illumination of the surface under investigation. In this paper, the Specim IQ has been used to detect the UV-induced luminescence with particular attention to the orange-red region of the visible range of the electromagnetic spectrum (≈ 550–730 nm). Due to the unconventional use of the hyperspectral camera, the experimental set-up has two differences with respect to the configurations used for reflectance measurements: (i) in the illumination system the halogen lamps have been substituted by UV emitters and (ii) the detector along with the sample have been shielded as much as possible from eventual undesired illumination coming from the UV sources or the environment. The former condition has been achieved by employing two professional ultraviolet lamps (3 Watt led lamps with peak emission at 365 nm), the latter acquiring the data in a dark room (for the ad hoc colour drafts, powders, and roots) or darkening the acquisition area with black curtains (for the *Quarto Stato*, the wooden palette and the Pellizza’s colour drafts). For what concerns the geometry of the set-up, the two lamps were placed on the sides of the investigated surface with a tilt of about 45 degrees to optimise the irradiation by limiting the phenomena of specular reflection with the additional precaution of managing the working distance based on the features of the sample under investigation. Due to the pictorial technique employed by Pellizza da Volpedo when he realised the painting, it is crucial to maintain the distance between the camera and the surface of *Quarto Stato* within 30 cm in order to be able to potentially distinguish the brush strokes^25^. Finally, since the physical origin of UV luminescence differs from that of reflectance, the default spectra normalisation with respect to the white reference used for reflectance measurements does not longer makes sense, and the data analysis has been performed employing the raw data.

### Data management and analysis

The management and analysis of the data have been performed exploiting codes home-developed in Matlab (MATLAB R2020b Natick, Massachusetts). In summary, the hyperspectral data are ordered and stored into variables designed to be managed by a two-steps algorithm, which parts are briefly called classifier and mapper, the former extracts the characteristic spectra of the selected areas of the hypercube, the latter evaluates the similarity between the data sets and the characteristic spectra by means of spectral correlation maps. Before being passed to the algorithm, the raw data undergo a pre-processing protocol: first, the spectra are smoothed by a sliding window mean filter, and then they are normalised to guarantee that each curve subtends an area equal to 1.

### The classifier

The spectra used as endmembers in the mapping function are obtained by clustering and classifying data from user-selected areas on the image and identified as significant: the user draws the desired number of polygons (also called Regions of Interest, ROIs) on RGB images representing the surface monitored by the hyperspectral camera, then the classifier evaluates the features of each spectrum corresponding to a pixel inside the polygon and by a custom support vector machine (SVM) creates clusters of spectra (pixels) named classes from which a single representative spectrum is extracted; this average spectrum can be used as endmember by the mapper. The SVM is based on the evaluation of 16 different distances between the vectors of the ROI generating distance maps which are then normalized between 0 and 1: only the maps, at least 3, which have a sufficient spread, evaluated by the algorithm, are therefore considered significant. Clustering occurs, pixel by pixel, by comparing the vectors deriving from the maps and finally, if the elements in a class verify a criterion decided by the user, then the specific class is considered significant for that ROI and the average spectrum from the elements of the class will be considered the characteristic spectrum of that class. The number of significant classes depends on the area of hypercube delimited by each ROI and it is also evident that the smaller the number of materials present in the selection, the smaller the number of characteristic spectra.

### The mapper

The Spectra Correlation Mapper, SCM, is a criterion of similarity between vectors of *m* components (hypervectors when *m* > 3) introduced as an evolution of the Spectra Angle Mapper, SAM, where the vectors are centred around their average value^20^:

1 $$SAM: \alpha =\frac{\mathrm{arccos}\sum \mathrm{XY}}{\sqrt{\sum ({X)}^{2}\sum {(Y)}^{2}}}$$

2 $$SCM: R=\frac{\sum (X-\underline{X})(Y-\underline{Y})}{\sqrt{\sum {(X-\underline{X})}^{2}\sum {(Y-\underline{Y})}^{2}}}$$

where SCM distinguishes between negative and positive correlation. In these expressions, one of the two hypervectors, *X* or *Y* indifferently, plays the role of the endmember while the other is each time one spectrum of the considered dataset; *R* ranges between − 1 and 1 and represents the degree of similarity between *X* and *Y*. The correlation maps reported in the figures of this paper have been obtained using the characteristic spectra obtained by the classifier as end members. Based on the options offered by the mapper^26^, the evaluation of the maps has been performed on the first derivative of the spectra in a range useful to focus on the UV-induced luminescence of red lakes: 550–730 nm; the wavelengths outside this range have been not accounted for the analysis. This choice together with the pre-processing guarantee some advantages: (i) the smoothing limits the effect of the white noise due to the detector, (ii) the normalisation together with the derivative reduces the dependence of the data on the intensity of the signal lowering the effects of small differences in the illumination of the area of the surface under investigation, (iii) the 550–730 nm range is focused on the region of the electromagnetic spectrum were red lakes are expected to emit luminescence and, simultaneously, allows to exclude those wavelengths affected by the reflection of the excitation source. The main disadvantage is the impossibility for the mapper to distinguish areas with differences in emission intensity but with the same spectral characteristics, this means that it cannot discriminate areas in which the only difference is given by the concentration of the same luminescent pigment.

### Supplementary Information

Supplementary Information.

## Data Availability

The datasets generated during and analysed during the current study are available from the corresponding author on reasonable request.

## References

[1] Li Y, Jia Y, Cai X, Xie M, Zhang Z (2022). Oil pollutant identification based on excitation-emission matrix of UV-induced fluorescence and deep convolutional neural network. Environ. Sci. Pollut. Res..

[2] Zeskind B (2007). Nucleic acid and protein mass mapping by live-cell deep-ultraviolet microscopy. Nat. Methods.

[3] Cerovic ZC, Samson G, Morales F, Tremblay N, Moya I (1999). Ultraviolet-induced fluorescence for plant monitoring: Present state and prospects. Agronomie.

[4] Huang Z, Omwange KA, Saito Y, Kuramoto M, Kondo N (2023). Monitoring strawberry (Fragaria × ananassa) quality changes during storage using UV-excited fluorescence imaging. J. Food Eng..

[5] Picollo M, Stols-Witlox M, Fuster López L (2020). UV–Vis luminescence: imaging techniques. Conservation.

[6] Bonizzoni L (2014). A multidisciplinary materials characterization of a Joannes Marcus viol (16thcentury). Herit. Sci..

[7] Fiocco G (2018). Approaches for detecting madder lake in multi-layered coating systems of historical bowed string instruments. Coatings.

[8] Cairns LK, Forbes PBC (2020). Insights into the yellowing of drying oils using fluorescence spectroscopy. Herit. Sci..

[9] Dondi P (2017). Automatic analysis of UV-induced fluorescence imagery of historical violins. J. Comput. Cult. Herit..

[10] Clementi C (2012). Photoluminescence properties of zinc oxide in paints: A study of the effect of self-absorption and passivation. Appl. Spectrosc..

[11] Gonzalez V (2017). Revealing the origin and history of lead-white pigments by their photoluminescence properties. Anal. Chem..

[12] Comelli D (2011). Insights into Masolino’s wall paintings in Castiglione Olona: Advanced reflectance and fluorescence imaging analysis. J. Cult. Herit..

[13] Verri G, Clementi C, Comelli D, Cather S, Piqué F (2008). Correction of Ultraviolet-induced fluorescence spectra for the examination of polychromy. Appl. Spectrosc..

[14] Bonizzoni L, Caglio S, Galli A, Lanteri L, Pelosi C (2023). Materials and technique: The first look at Saturnino Gatti. Appl. Sci..

[15] Lange, R., Zhang, Q., & Liang, H. Remote multispectral imaging with PRISMS and XRF analysis of Tang tomb paintings. In *Proceedings of the SPIE 8084, O3A: Optics for Arts, Architecture, and Archaeology III*, 80840Y; 10.1117/12.890973 (2011).

[16] Amadori ML (2015). Invasive and non-invasive analyses for knowledge and conservation of Roman wall paintings of the Villa of the Papyri in Herculaneum. Microchem. J..

[17] Behmann J (2018). Specim IQ: Evaluation of a new, miniaturized handheld hyperspectral camera and its application for plant phenotyping and disease detection. Sensors.

[18] Bedini, E. *Preparazione della lacca di robbia*. https://emanuelebedini.wordpress.com/2016/08/26/preparazione-della-lacca-di-robbia/ (2016)

[19] Mounier A, Belin C, Daniel F (2011). Spectrofluorimetric study of the ageing of mixtions used in the gildings of mediaeval wall paintings. Environ. Sci. Pollut. Res..

[20] De Carvalho Jr, O. A., & Meneses, P. R. Spectral correlation mapper (SCM): An improvement on the spectral angle mapper (SAM). In *Summaries of the 9th JPL Airborne Earth Science Workshop*, Vol. 9. JPL Publication (2000).

[21] Poldi G. Ricostruire la tavolozza dei divisionisti mediante spettrometria in riflettanza: Pellizza da Volpedo, le prove di colore e il quarto stato, *Il Colore dei Divisionisti*. *Tecnica e teoria, analisi e prospettive di ricerca*. (Ed Scotti Tosini, A.), 119–134, (Ass. Pellizza da Volpedo, 2007). ISBN 9788890021688.

[22] Bonizzoni L (2021). Balla’s bouquet: A pigment study for flowers and lights. J. Cult. Herit..

[23] Bonizzoni L, Capurro R, Galli A, Taccola G (2020). Ottica divisionista: Armonia parlante in Quarto Stato. Nel Quarto Stato.

[24] Previati G (2012). Principi Scientifici del Divisionismo.

[25] Hartley, R. I., Gupta, R. Linear pushbroom cameras. In *Computer Vision—ECCV'94. ECCV 1994* (Ed. Eklundh, J. O.), Lecture Notes in Computer Science, 800. 10.1007/3-540-57956-7_63 (1994).

[26] Caccia M (2021). Applying hyperspectral reflectance imaging to investigate the palettes and the techniques of painters. J. Vis. Exp..

