# Supplementary material for: Objective interpretation of ultraviolet-induced luminescence for characterizing pictorial materials
Source: Sci Rep. 2023 Nov 19;13:20240. doi: 10.1038/s41598-023-47006-x (PMC10658075; doi:10.1038/s41598-023-47006-x)
Supplement: Supplementary file 1 — Supplementary Information. [file 41598_2023_47006_MOESM1_ESM.docx]

**Quantitative interpretation of Ultraviolet-induced luminescence for characterizing pictorial materials**

M. Caccia^1^, S. Caglio^2*^, A. Galli^2,1^

1 IBFM-CNR, Via Fratelli Cervi 93, Segrate (MI), Italy
2 Dipartimento di Scienza dei Materiali, Università degli Studi di Milano-Bicocca, Via Roberto Cozzi 55, Milano, Italy
*simone.caglio@unimib.it

# **What happens on UVL signal when samples exposed to light?**

The cardboard with the ad-hoc drafts (Figure 1, panel d) has been placed in a solar simulator and irradiated by an increasing dose of light to mimic the effect of different periods of sunlight exposure on a painted surface. It is reasonably to suppose that ancient paintings have been exposed, directly or indirectly, to a significant amount of light that could potentially modify the luminescence emission of their pigments. In order to create a reliable spectra database, the pigments used as reference must be put in similar conditions; this task has been performed by means of the solar simulator and Figure S9 summarises the obtained results. The panels show that, independently from the binder, the luminescence signals experience an initial growth to reach a stable plateau once subjected to an amount of light comparable to that they would suffer if exposed to sunlight for a continuative period of 12 months. The emission intensity does not show significant variations with further increasing of light exposure, probably also due to the low UV-induced luminescence intensity. The results resumed by Figures 2 and S9 are extremely important; the former demonstrates that it is possible to associate with madder lakes a significant luminescence spectrum and that, even if the madder luminescence is almost independent of the binders, when a significant, even small, difference is present (as in the case of Z1 and Z2 mixed to AG), it can be detected. Taken together, these facts allow us not only to qualitatively compare pictorial materials produced at different times (i.e., paintings, palettes or colour tubes belonged to the author within themselves or with the drafts prepared ad hoc in different periods of time) but also to consider the characteristic spectra derived from the hypercube as a real database that can be used to quantitatively identify the pigments employed by the masters.

**Solar Simulator**

On samples created in the laboratory, the ad hoc drafts, accelerated photo-oxidative ageing was carried out, in order to (i) investigate the effects of deterioration due to light exposure, which could also modify the emission of luminescence, in addition to the fading of colour and to (ii) put fresh paints in a condition similar to those use by Pellizza. A solar simulator from Honle UV technology (model SOL 500,) was used to irradiate the samples. To define the exposure times, the power absorbed per square metre by a surface exposed in a room with white walls, indirectly lighted by solar radiation coming from an unshielded window, on a sunny spring day was measured; from the results, it was calculated that 5 hours and 42 minutes of solar simulator correspond to one month of 24/7 natural irradiation. So, the ad hoc samples have been irradiated for a time equivalent to 1, 3 and 12 months of exposure to sunlight.


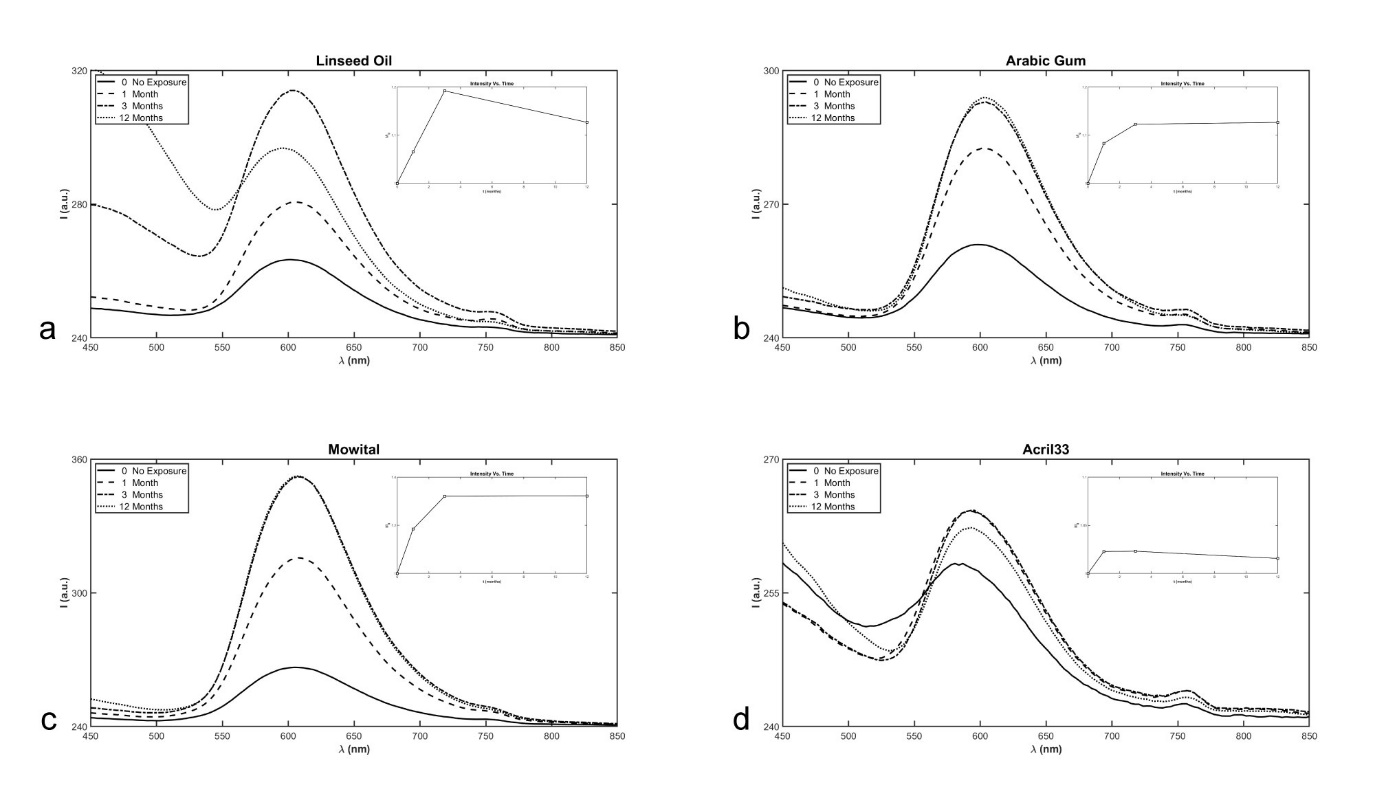
**Figure S9** – Variation of the intensity of emission of UV-induced luminescence compared to the equivalent time of exposure to the solar simulator. Curves for home-made madder lake with (a) linseed oil, (b) Arabic gum, (c) Mowital vinyl binder, (d) Acril 33 acrylic binder are presented.
